# Denture hygiene knowledge, cleaning practices, and denture stomatitis among primary care patients in Alkharj, Saudi Arabia: compliance predictors, barriers and educational preferences: a cross-sectional study

**DOI:** 10.3389/fmed.2026.1927858

**Published:** 2026-09-23

**Authors:** Rafif Alshenaiber

**Affiliations:** Prosthetic Dental Sciences Department, College of Dentistry, Prince Sattam bin Abdulaziz University, Al-Kharj, Saudi Arabia

**Keywords:** denture hygiene, denture stomatitis, edentulism, health equity, oral health compliance, oral-systemic health, primary care integration, Saudi Arabia

## Abstract

**Background:**

Denture wearers must maintain good denture hygiene, as oral health significantly impacts systemic health; yet denture hygiene practices remain poorly addressed within dental primary care facilities. Saudi Arabia's dental primary care system serves a demographically diverse population, including a large proportion of non-Saudi patients, creating a setting in which sociodemographic, linguistic, and economic factors intersect to shape the oral health behaviors of individuals who wear dentures.

**Objectives:**

To assess denture hygiene knowledge, cleaning behaviors, and denture stomatitis among denture wearers attending a single primary care setting in Alkharj, Saudi Arabia, and to identify behavioral predictors and patient-reported barriers to denture hygiene compliance.

**Methods:**

A cross-sectional study of 120 patients attending a dental primary care clinic was conducted. A questionnaire assessed knowledge, barriers, and educational preferences about oral/denture hygiene care. A clinical examination assessed prosthetic history and classified denture stomatitis using the Newton system. Statistical analyses included independent *t*-tests, one-way ANOVA, Pearson correlation, chi-square tests, and binary logistic regression (*p* = 0.05).

**Results:**

Only 24.2% of patients demonstrated adequate knowledge of oral/denture hygiene. In comparison, high-income patients had significantly higher mean knowledge scores than low-income patients (*p* < .001); in the adjusted multivariable model, income level and nationality were each independently associated with knowledge adequacy. Young females (age <50 years) showed significantly higher oral/denture hygiene compliance (*p* < 0.001). 63.3% of patients reported wearing their denture during sleep due to forgetfulness, and 65.8% suffer from denture stomatitis. Written instructions were preferred by 51.7% of patients, and teaching hospitals were the main source of oral/denture care instructions. The most commonly mentioned barrier to receiving comprehensive oral/denture hygiene instruction was “dentists have no time to give instructions” (40.8%).

**Conclusion:**

Denture hygiene compliance is critically low among patients attending primary care settings in Alkharj, Saudi Arabia. Non-Saudi nationality, low income, greater tooth loss, and male sex are independent correlates of non-compliance. Forgetfulness regarding nighttime removal and social embarrassment about public denture cleaning represent prevalent behavioral barriers to non-compliance. The strong preference for written educational materials and the high prevalence of denture stomatitis collectively underscore the clinical and public health imperative for integrating brief, culturally adapted, written denture hygiene counseling into routine primary care in Saudi Arabia.

## Introduction

Oral health is a major component of overall health, wellbeing, and quality of life (1). Removable dentures with or without implants remain among the most widely utilized oral rehabilitative prostheses, particularly in aging populations (2). However, without adequate maintenance, removable dentures are significant microbial reservoirs (3, 4). They facilitate the accumulation of polymicrobial oral biofilm harboring pathogenic microorganisms, including Candida albicans, Staphylococcus aureus, and anaerobic respiratory pathogens (5–7).

Nighttime denture wearing and poor oral/denture hygiene have been independently associated with multiple oral and systemic complications (8, 9). The most immediate sequela of poor hygiene affecting oral health is denture stomatitis. It is estimated that 20%–67% of denture wearers are affected by denture stomatitis (10, 11). Poor denture care can increase the potential for developing eating and swallowing disorders in denture wearers (12). The systemic implications of poor denture hygiene increase the risk of aspiration pneumonia, infective endocarditis, and septic meningitis due to harmful biofilm formation (5, 9, 13, 14). Moreover, oral Candida colonization facilitated by denture biofilm has been linked to diabetes mellitus (15, 16). Despite this abundant literature, denture hygiene assessment and education remain largely confined to dental schools and are rarely integrated into primary care settings (17, 18). Improper denture hygiene education, varied cleaning methods, and cleaning agents may also contribute to inadequate patient education (17, 19).

In Saudi Arabia, the primary healthcare system serves a large and diverse population. The recent report by the Saudi General Authority for Statistics found that Saudi Arabia is home to approximately 15.7 million non-Saudi expatriate residents (20). Many non-Saudi employees experience low income and restricted access to dental care, coupled with lower levels of education and possible language obstacles (21). These factors may compound to produce a high-risk oral health profile (22). However, all non-Saudi residents with a working permit are eligible to receive free dental care in the Ministry of Health in Saudi Arabia primary care clinics. Moreover, several teaching dental hospitals in Saudi Arabia provide free dental treatment to non-Saudi residents (23).

Several recent studies have examined denture care behaviors in comparable populations, including a single-institution cross-sectional study of denture care attitudes, hygiene levels, and oral mucosal lesions (24) a multicenter study of behaviors, hygiene habits, and sources of care among removable denture wearers (25) and an assessment of denture care and oral health-related quality of life among complete denture wearers in the Eastern Province of Saudi Arabia (26). While these studies confirm a substantial need to enhance denture hygiene routines, none was conducted within a primary care framework, systematically assessed patient-reported barriers to receiving hygiene instruction, or captured a demographically diverse population inclusive of both Saudi and non-Saudi residents. The present study addresses this gap by evaluating denture hygiene knowledge, compliance, and stomatitis within a primary care setting while explicitly assessing behavioral barriers and educational-format preferences across a diverse patient population.

The present study therefore aimed to determine oral and denture hygiene knowledge, cleaning frequency, methods, and denture stomatitis among primary care attendees in Alkharj, Saudi Arabia, based on: (1) sociodemographic factors; (2) prosthetic history; and (3) systemic disease, and to identify behavioral predictors of non-compliance and evaluate patient-reported barriers and educational format preferences, to inform the integration of denture hygiene counseling into routine primary care encounters.

## Materials and methods

### Ethics

Ethical approval was obtained from the Standing Committee of Bioethics Research (SCBR) of Prince Sattam bin Abdulaziz University, Deanship of Scientific Research, with Reference: SCBR-277/2024. Written informed consent was secured from all participants prior to data collection. The study was conducted in accordance with the Declaration of Helsinki.

### Study design and setting

This cross-sectional observational study was conducted between April 2024 and April 2026 at dental interns' primary care clinics affiliated with the College of Dentistry, Prince Sattam bin Abdulaziz University, Alkharj, Saudi Arabia. Within the university, these dental intern clinics are the first-contact point for routine and walk-in dental care for the general population of Alkharj city. Attendance in this clinic does not require a prior specialist referral and is therefore considered part of the primary dental care rather than a tertiary referral clinic. Intern clinics were selected as the recruitment setting to capture a community-representative sample of denture wearers who attend dental clinics seeking dental care.

### Participants and sampling

Adults aged 18 years or above wearing a complete or conventional partial removable dental prosthesis for a minimum of six months were eligible. Exclusion criteria comprised: severe cognitive impairment precluding informed consent, and refusal to participate. An *a priori* sample size estimate was calculated using Cochran's formula for estimating a proportion in a cross-sectional design at a 95% confidence level (Z = 1.96), *p* = 0.50, and a margin of error of e = 0.09; this yielded an initial estimate of n₀ of about 119. This estimate was further adjusted for the population of Alkharj governorate (*N* = 373,177) (27) using the finite population correction:

$$n=\frac{n_{0}}{1+\;(n^{0}-1/N)}$$

which yielded an adjusted minimum required sample of approximately 119 participants; because N is very large relative to n₀, the finite-population correction altered the required sample size negligibly. Consecutive sampling was employed during regular clinic hours. A total of 148 patients were screened during the study period; 28 were excluded (14 had no current denture, 8 wore implant-supported prostheses only, 6 declined to participate), yielding a final sample of 120 patients which met this *a priori* target (Figure 1).

**Figure 1:**
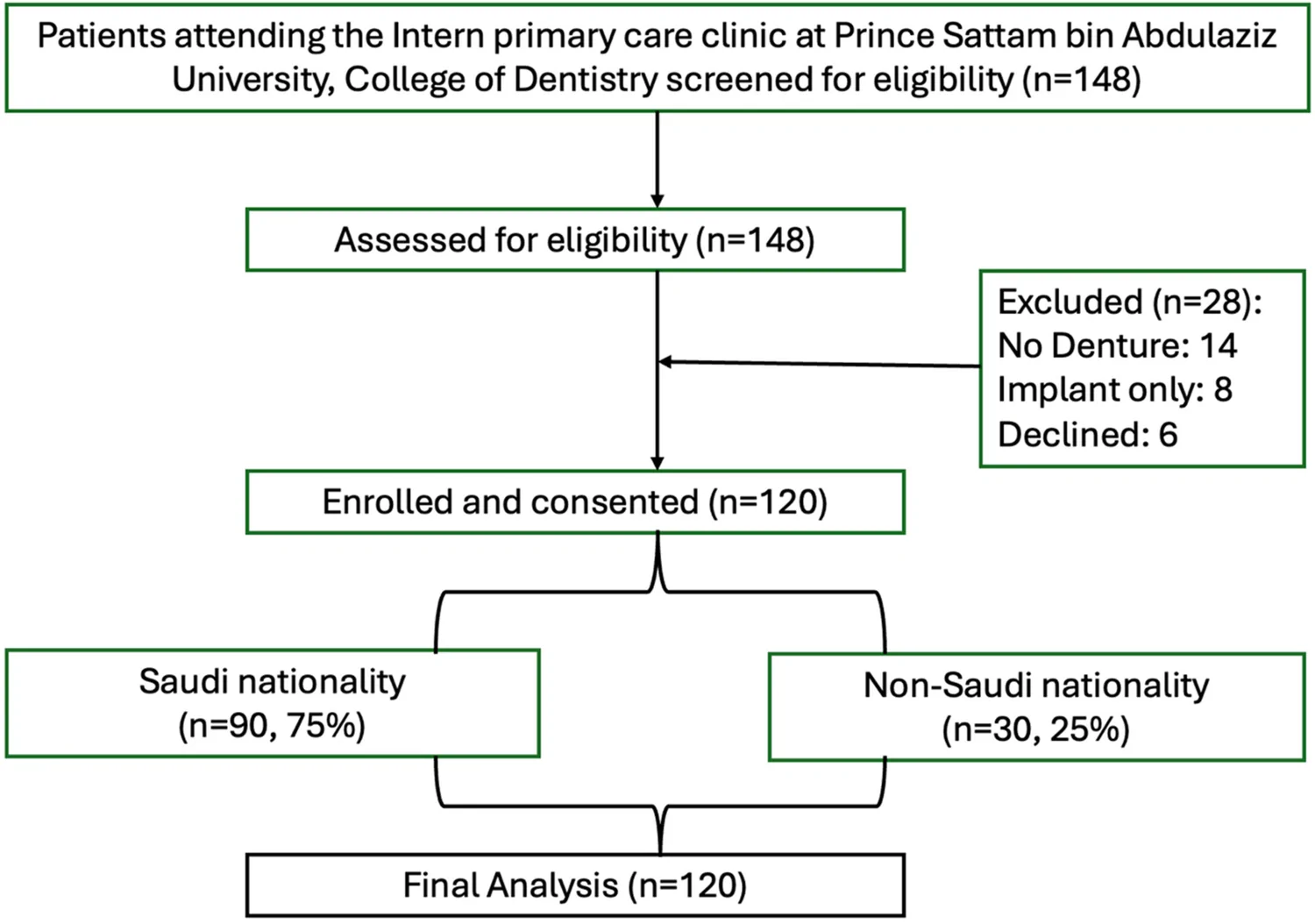
STROBE flow chart of participant recruitment, screening, and enrollment.

### Data collection

A structured questionnaire comprising five domains was adminestered to participants: (i) sociodemographic characteristics (nationality (Saudi vs. non-Saudi), age, gender, native language (Arabic vs. non-Arabic speaking), education level (none/primary, secondary, or university/postgraduate), income level (low, moderate, high)); (ii) Systemic disease status was self-reported and categorized as: none, hypertension, diabetes mellitus, combined diabetes and hypertension (DM + HTN), or other systemic conditions. (iii) Oral hygiene knowledge validated questionnaire adapted from literature (28, 29); evaluated based on: (last dental visit, length of denture wear, self-reported compliance behaviors [proper cleaning of denture, cleaning frequency (cleaning episodes per day: 0, 1, 2, or 3+), cleaning method (no cleaning, water rinse only, soak only, brushing only, combined brushing and soaking, combined brushing/soaking/ultrasonic), nighttime denture removal, receipt of denture hygiene care instruction). (iv) Patient-reported barriers to receiving oral/denture hygiene care instructions, partially adopted from a similar study (30). (v) Educational format preferences to receive oral/denture hygiene care instructions.

Oral/denture hygiene knowledge was defined as adequate when the knowledge score was ≥5 out of 6 from the oral hygiene knowledge validated questionnaire. The questionnaire was adapted from previously published denture-hygiene survey instruments (28, 29). Questionnaire adaptation involved content review by the investigating prosthodontist (Arabic native speaker, qualified in the United Kingdom) for content validity and rewording into the Arabic language before data collection. The Arabic-language version was then reviewed and approved for content validity by a panel of six prosthodontics consultants, all native Arabic speakers with English as a second language. A pilot administration in the present sample was not performed, and item-level responses were recorded only as an aggregate knowledge score rather than as individual item scores. No reliability analysis was performed on the adapted knowledge scale in this sample.

The questionnaire was administered as a self-completed electronic form (Google Forms) presented to each participant on a tablet. For participants unable to read, the device's text-to-speech function was activated so that each item could be heard rather than read, in formal Arabic. The nine non-Arabic-speaking participants completed the same Arabic-language form with the assistance of a translation tool (Google Translate). A research nurse who speaks Arabic and English was available to help participants with the technical operation of the tablet (navigating between items and tapping response options) but did not read, interpret, or influence the content of participants' answers.

A prosthodontist consultant with 15 years of clinical experience performed a clinical examination of the missing teeth pattern, prosthesis type, and prosthetic-bearing mucosa, and denture stomatitis was classified using the Newton grading system (31). Intra-examiner reliability of denture stomatitis grading was assessed by having the examiner re-grade a set of 25 clinical photographs of denture-bearing mucosa approximately two weeks after the initial assessment; this reliability exercise was completed before patient recruitment for the present study. Agreement between the two grading occasions was 88% (22/25 photographs), with a linear-weighted Cohen's kappa of 0.81.

### Statistical analysis

Data were analyzed using IBM SPSS Statistics version 26.0 (IBM Corp., Armonk, NY, USA). The normality of continuous variables (knowledge score, oral health-related quality of life score, age, and total missing teeth) was assessed using the Shapiro–Wilk test. Binary logistic regression was performed to identify independent predictors of adequate oral/denture hygiene knowledge, with nationality, age, sex, education, income level, and last dental visit entered as covariates. Multicollinearity among predictors was assessed using variance inflation factors, and model calibration was evaluated using the Hosmer-Lemeshow goodness-of-fit test; overall model fit was summarized using McFadden's pseudo-R². Descriptive statistics summarized frequencies, percentages, means, and standard deviations. Between-group knowledge score comparisons were performed using independent-samples *t*-tests and one-way ANOVA (post-hoc Tukey correction). Pearson correlation examined the linear association between missing teeth and knowledge score. Chi-square tests assessed categorical associations. Statistical significance was set at *p* = 0.05.

## Results

### Sociodemographic and clinical characteristics

The study sample comprised 120 denture-wearing adults aged 35–77 years. Ninety participants (75%) were Saudi nationals; of the 30 non-Saudi participants, 21 (70%) were Arabic-speaking, and 9 participants (30%) communicated in other languages. Full sociodemographic data are presented in Table 1.

**Table 1:** Sociodemographic and systemic health characteristics of participants stratified by nationality.

Characteristic	Saudi (*n* = 90)	Non-Saudi (*n* = 30)	Total (*n* = 120)
Age, mean ± SD (years)	57.2 ± 13.8	58.2 ± 14.9	57.5 ± 14
Range (years)	35–77	35–77	35–77
Sex, *n* (%)			
Female	46 (51.1%)	15 (50%)	61 (50.8%)
Male	44 (48.9%)	15 (50%)	59 (49.2%)
Language, *n* (%)			
Arabic-speaking	90 (100%)	21 (70%)	111 (92.5%)
Non-Arabic-speaking	0 (0%)	9 (30%)	9 (7.5%)
Education level, *n* (%)			
None/Primary	18 (20%)	15 (50%)	33 (27.5%)
Secondary	26 (28.9%)	6 (20%)	32 (26.7%)
University/Postgraduate	46 (51.1%)	5 (16.7%)	51 (42.5%)
Monthly income level, *n* (%)			
Low (<SAR 4,000)	8 (8.9%)	14 (46.7%)	22 (18.3%)
Moderate (SAR 5,000–10,000)	23 (25.6%)	10 (33.3%)	33 (27.5%)
High (>SAR 10,000)	59 (65.6%)	6 (20%)	65 (54.2%)
Systemic disease, *n* (%)			
None	21 (23.3%)	9 (30%)	30 (25%)
Diabetes mellitus	27 (30%)	6 (20%)	33 (27.5%)
Hypertension	16 (17.8%)	5 (16.7%)	21 (17.5%)
Diabetes + Hypertension	16 (17.8%)	3 (10%)	19 (15.8%)
Other	10 (11.1%)	7 (23.3%)	17 (14.2%)

The patients' prosthetic data are summarized in Table 2 and Figures 2,3.

**Table 2:** Prosthetic characteristics, tooth loss patterns, and dental attendance of study participants.

Variable	*n* (%)
Denture type	
Complete denture (age 64–77 years)	55 (45.8%)
Partial denture (age 35–63 years)	65 (54.2%)
Most frequently missing teeth (partial denture wearers only)	
First and second molars	52 (80%)
Premolars	9 (13.8%)
Anterior teeth	4 (6.2%)
Last dental visit	
Less than 6 months	22 (18.3%)
6–12 months	29 (24.2%)
1–2 years	33 (27.5%)
More than 2 years	36 (30%)

**Figure 2:**
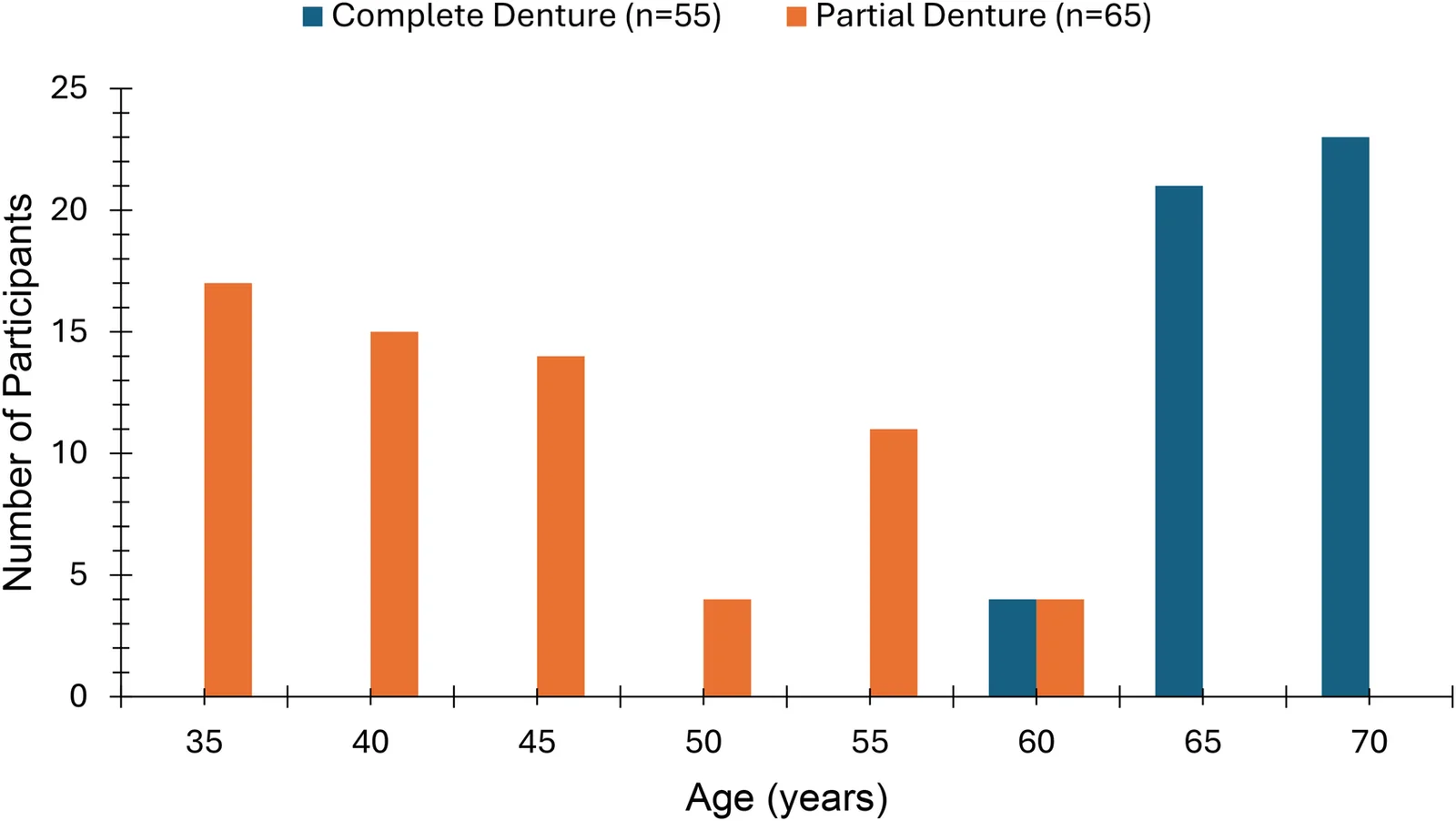
The Age distribution by denture type.

**Figure 3:**
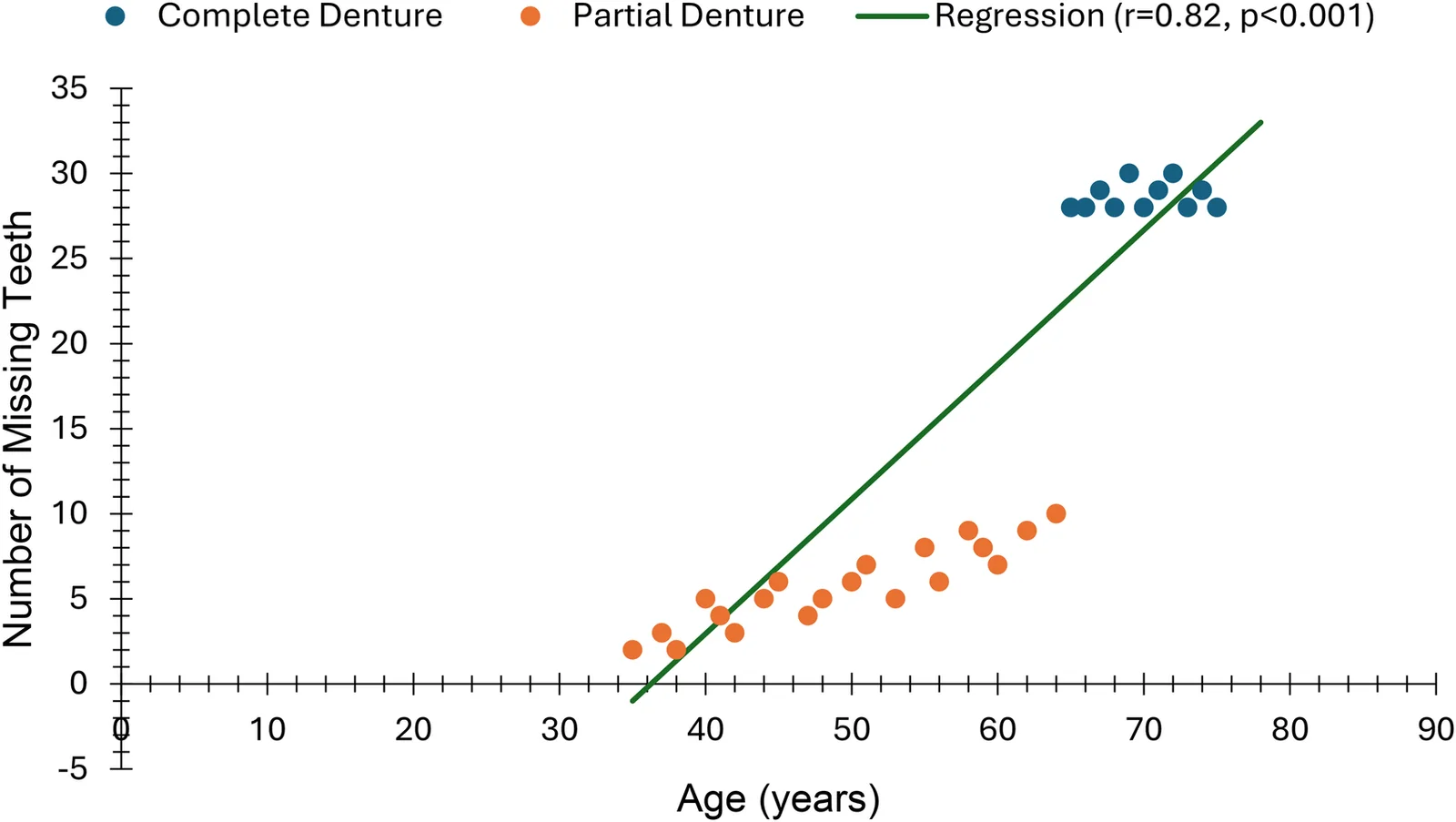
The association between patients' age and number of missing teeth.

Shapiro–Wilk testing on all continuous variables showed that knowledge score (W = 0.993, *p* = 0.809) did not deviate from normality, while age (W = 0.911, *p* < 0.001) and total missing teeth (W = 0.730, *p* < 0.001) departed from normality.

### Oral/denture hygiene knowledge

A total of 29 participants (24.2%) achieved an adequate oral/denture hygiene knowledge score (≥5/6), indicating that more than three-quarters of the study population (75.8%) lacked adequate knowledge of appropriate oral and denture hygiene practices. Chi-square analysis confirmed a significant association between nationality and knowledge adequacy (*p* = 0.005); Saudi nationals were more likely than non-Saudi patients to demonstrate adequate denture hygiene knowledge. Knowledge also varied by age, education level, and income level.

With respect to prosthetic history, recency of the last dental visit and the method and frequency of denture cleaning were significant predictors of adequate oral/denture hygiene knowledge. Nighttime denture removal was also a significant behavioral predictor. In contrast, no significant association was observed between systemic disease status and adequate knowledge. Table 3 summarizes the association between adequate oral/denture hygiene knowledge and key sociodemographic, behavioral, and clinical factors.

**Table 3:** Association between adequate oral/denture hygiene knowledge and key sociodemographic, behavioral, and clinical factors.

Factor	Category	Adequate Knowledge (%)	*P* Value
Nationality	Saudi vs. non-Saudi	31.1% vs. 3.3%	0.005
Age/sex	Young females (<50y) vs. all others	Significantly higher (exact % not reported)	<0.001
Education level	Primary/Secondary/University/Postgraduate	4.5%/25.0%/31.2%/47.4%	<0.001
Income level	Low/Moderate/High	0%/13.3%/74.3%	<0.001
Last dental visit recency	≤6 mo/6–12 mo/1–2 y/ > 2 y	63.6%/34.5%/12.1%/2.8%	<0.001
Cleaning method	No cleaning method used	0% (adequate) vs. 24.2% (inadequate)	<0.001
Cleaning frequency	Zero daily cleaning episodes	0% (adequate) vs. 34.1% (inadequate)	<0.001
Nighttime denture removal	Removed vs. not removed during sleep	50.0% vs. 9.2%	<0.001
Systemic disease status	Any systemic disease vs. none	Not significant	0.522

### Predictors of adequate oral/denture hygiene knowledge

The income level (95% CI: 3.66–13,301.45, *p* = 0.002) and recency of last dental visit (95% CI: 3.75–532.57, *p* = 0.0005) were the strongest independent correlates of adequate knowledge, while nationality (95% CI: 0.96–2,101.64, *p* = 0.054) showed a borderline association; age (95% CI: 0.84–1.04, *p* = 0.206), sex (95% CI: 0.72–12.03, *p* = 0.131), and education type (95% CI: 0.01–3.03, *p* = 0.225) were not independent predictors once other covariates were accounted for. Variance inflation factors for all predictors were below 5 (range 1.02–4.98), indicating no problematic multicollinearity. The Hosmer-Lemeshow test indicated good model calibration (*χ*^2^ = 3.37, df = 8, *p* = 0.909), and the model demonstrated strong discriminative performance (McFadden's pseudo-R^2^ = 0.642).

### Frequency of denture cleaning

Nearly three-quarters of patients (74.2%) cleaned their dentures once daily or less, indicating widespread suboptimal practice in this cohort. Non-Saudi patients were more likely to report no daily cleaning and less likely to clean three times daily. Age and gender were not significantly associated with cleaning frequency, but education and monthly income were.

Last dental visit recency was significantly associated with cleaning frequency of dentures. Denture type was not significantly associated with cleaning frequency, although partial denture wearers demonstrated greater twice-daily cleaning rates. Moreover, systemic disease status was not significantly associated with daily denture-cleaning frequency, with the full frequency distribution and its associations with sociodemographic and clinical factors summarized in Table 4.

**Table 4:** Association between denture cleaning frequency and sociodemographic and clinical factors.

Factor	Finding	*P* Value
Overall distribution	74.2% of patients cleaned their dentures once daily or less (*n* = 120)	–
Nationality	Non-Saudi patients more often reported no daily cleaning (43.3% vs. 20.0%) and less often cleaned 3x/day (0% vs. 6.7%)	0.032
Language	Similar but non-significant pattern (small non-Arabic-speaking subgroup, *n* = 9)	0.153
Age	Not significant	0.129
Gender	Not significant	0.378
Education level	Non-cleaning highest with primary education (50%), lowest with postgraduate (10.5%)	0.021
Income level	Non-cleaning highest in low-income group (45.5%); 67.6% of high-income patients cleaned ≥ once daily	0.001
Last dental visit recency	≤6 mo: highest twice-daily cleaning (31.8%), no zero-cleaning cases; >2 y: highest zero-cleaning (41.7%)	0.048
Denture type	Not significant; partial-denture wearers showed higher twice-daily cleaning (26.2% vs. 12.7%)	0.333
Systemic disease status	Not significant; hypertension-only patients showed highest ≥ once-daily cleaning (90.5%)	0.246

### Denture cleaning method

Cleaning method was not significantly associated with nationality, language, or age. Brushing alone was the most common cleaning method among patients aged 40–49 years and declined with age, while passive methods (soaking, water rinse) became more common in older patients. Education and income level were likewise not significantly associated with cleaning method, although low-income participants showed the highest proportion of water rinse alone.

Regarding prosthetic history, last dental visit was a significant predictor of cleaning method choice. Patients seen within six months were most likely to use a combined brushing-and-soaking regimen, while those with a visit gap exceeding two years were most likely to use water rinse alone or brush only, suggesting a gradual decline in denture-care practices with increasing disengagement from dental care. Denture type did not significantly affect the choice of cleaning method. Similarly, cleaning method varied across systemic disease categories, but the differences were not statistically significant. Table 5 summarizes the association between denture cleaning method and key sociodemographic and clinical factors.

**Table 5:** Denture cleaning frequency and method: association with sociodemographic and clinical factors.

**Factor**	**Finding**	***P* Value**
Nationality	Not significant	0.510
Language	Not significant	0.068
Age	Not significant; brushing-only most common at 40–49y (33.3%), declining with age (13.8% at ≥70y)	0.155
Gender	Not significant; females more often used brush + soak (24.6% vs. 16.9%), males more often water rinse (28.8% vs. 19.7%)	0.389
Education level	Not significant	0.488
Income level	Not significant; low-income patients showed highest water-rinse-only use (45.5%)	0.408
Last dental visit recency	Significant; ≤6 mo: highest brush + soak use (45.5%); >2 y: highest water-rinse-only (36.1%) or brush-only (33.3%)	0.003
Denture type	Not significant; complete-denture wearers favored water rinse (29.1%), partial-denture wearers favored brushing alone (26.2%)	0.142
Systemic disease status	Not significant; diabetic patients most often used water rinse (39.4%); DM + HTN patients showed diverse use; other conditions most often used brushing only (31.2%)	0.271

### Denture stomatitis

Clinically detectable denture stomatitis was present in 65.8% of participants (79/120). Newton Type II (diffuse erythema) was the most prevalent grade (35.8%), followed by Type I (21.7%) and Type III (8.3%). All Type III cases occurred among complete denture wearers who did not comply with denture removal during sleep. The proportion of non-compliant participants increased with stomatitis severity, suggesting a clinically graded relationship between non-compliance and the severity of mucosal inflammation.

Nationality was significantly associated with denture stomatitis severity, whereas the association between language and stomatitis severity was not significant. Mean age increased progressively and substantially with stomatitis severity; Bonferroni-corrected *post-hoc* comparisons confirmed that participants with no stomatitis were significantly younger than those with Type II (*p* < 0.001) or Type III (*p* < 0.001) disease, and that Type I patients were significantly younger than Type II patients (*p* = 0.018). Gender, education level, and monthly income showed no significant association with denture stomatitis type.

Recency of the last dental visit did not significantly predict denture stomatitis type, whereas denture type was a significant predictor of stomatitis severity. No significant association was found between denture cleaning method and stomatitis type; however, participants who cleaned their dentures with “nothing” or “soak only” had lower proportions free of stomatitis (31.8% and 33.3%, respectively). By contrast, cleaning frequency showed a significant and clinically meaningful association with the presence of denture stomatitis, with prevalence decreasing progressively as cleaning frequency increased.

Among the 79 patients with denture stomatitis, the distribution of Newton classification was further examined in relation to nighttime denture-wearing habits. Failure to remove the denture during sleep was significantly associated with stomatitis type: half (50%) of participants who removed their dentures during sleep had no stomatitis, compared with only 25.0% of those who habitually did not. Most strikingly, all 10 cases of severe Type III stomatitis in the entire sample occurred exclusively among participants who continued wearing their dentures during sleep. Systemic disease status was also significantly associated with denture stomatitis type. Table 6 summarizes the associations between denture stomatitis and key sociodemographic, behavioral, and clinical factors.

**Table 6:** Association between denture stomatitis and key sociodemographic, behavioral, and clinical factors.

Factor	Finding	*P* Value
Nationality	Saudi patients predominantly Type II (55.9%), rarely Type III (6.8%); non-Saudi patients showed higher Type III (30.0%) and lower Type I (20.0% vs. 37.3%)	0.020
Language	Not significant	0.552
Age	Mean age rose progressively with severity (48.4 → 54.5 → 65.3 → 69.2 years, no stomatitis → Type III)	<0.001
Gender	Not significant	0.168
Education level	Not significant	0.552
Income level	Not significant	0.438
Last dental visit recency	Not significant	0.493
Denture type	Complete-denture wearers predominantly Type II (64.0%, 20.0% Type III); partial-denture wearers predominantly Type I (62.1%), 0% Type III	<0.001
Cleaning method	Not significant	0.541
Cleaning frequency (stomatitis presence)	Stomatitis prevalence decreased progressively with increasing cleaning frequency (exact per-category percentages not restated in text)	0.006
Newton type by cleaning frequency	No significant pattern identified	0.523
Nighttime denture removal	50.0% of patients who reliably removed dentures during sleep had no stomatitis	0.002
Systemic disease status	Stomatitis prevalence markedly higher with combined diabetes and hypertension (89.5% combined Types I–III, 15.8% Type III) and hypertension alone (71.4% combined, 19.0% Type III)	0.032

### The behavioral predictors of non-compliance

A significant proportion of patients (63.3%) who wear dentures during sleep indicated that forgetfulness was the primary reason for this behaviour rather than deliberate decision-making. Additionally, 30% of patients reported feelings of embarrassment associated with the removal of dentures during sleep. Approximately 6.7% of participants expressed the belief that it was unnecessary to take out their dentures while sleeping.

Social embarrassment regarding public denture removal represents a significant psychosocial barrier to inter-meal and post-meal denture hygiene. Only 10% of patients stated that they would remove and clean their dentures in a public setting; conversely, 72 participants (60%) admitted to feeling embarrassed about the prospect of removing or cleaning their dentures in front of others. Furthermore, 22% of the patients felt it was unnecessary to remove their dentures for cleaning, and only 8% reported that they refrain from wearing their dentures in public.

### Patient-reported barriers and educational format preferences to integrate oral/denture hygiene instructions

Patients reported different sources of denture hygiene instructions; a detailed breakdown by nationality is presented in Table 7.

**Table 7:** Source of denture hygiene instructions by nationality.

Instruction Source	Overall (*n* = 120)	Saudi (*n* = 90)	Non-Saudi (*n* = 30)	*p*-value
Teaching Hospital	92 (76.7%)	71 (78.9%)	21 (70%)	0.312
Governmental Hospital	7 (5.8%)	6 (6.7%)	1 (3.3%)	0.517
Ministry of Health Clinic	6 (5%)	3 (3.3%)	3 (10%)	0.144
Private Clinic	5 (4.2%)	4 (4.4%)	1 (3.3%)	0.791
Cannot Recall	6 (5%)	6 (6.7%)	0 (0%)	0.180
Abroad	4 (3.3%)	0 (0%)	4 (13.3%)	<0.001*

Patients reported several barriers to receiving comprehensive oral/denture hygiene instructions. Table 8 presents the frequency of each barrier endorsed by patients.

**Table 8:** Frequency of barriers to receiving comprehensive oral and denture hygiene instructions (*n* = 120).

Barrier (Multiple Responses Permitted)	n	%	Rank
Dentists have no time to give instructions	49	40.8	1
Expensive appointments	48	40	2=
No perceived need for professional instructions	48	40	2=
Appointments are difficult to get	46	38.3	4
Time shortage (patient side)	42	35	5
Fear of dental appointments	34	28.3	6
Other	14	11.7	7

Table 9 presents barrier profiles among participants who received instructions from teaching hospitals vs. other settings.

**Table 9:** Barrier frequency by instruction source (teaching hospital vs. other settings).

Barrier	Teaching hospital (*n* = 92)—*n* (%)	Other settings (*n* = 28)—*n* (%)	*p*-value
Fear of dental appointments	28 (30.4%)	6 (21.4%)	0.344
Expensive appointments	36 (39.1%)	12 (42.9%)	0.726
Time shortage (patient side)	30 (32.6%)	12 (42.9%)	0.316
Dentists have no time to give instructions	39 (42.4%)	10 (35.7%)	0.519
Appointments are difficult to get	31 (33.7%)	15 (53.6%)	0.062
No perceived need for professional instructions	34 (37.0%)	14 (50.0%)	0.208
Other	9 (9.8%)	5 (17.9%)	0.248

Regarding educational preference for oral/denture hygiene instructions, 51.7% (62) of participants preferred written instructions, compared to 18.3% (22) who preferred verbal instructions alone, 24.2% (29) who preferred a combination of both, and 5.78% (7) who preferred video or digital formats.

## Discussion

This study investigated oral/denture hygiene knowledge, denture hygiene compliance, and denture stomatitis in primary care settings in Alkharj, Saudi Arabia, using a demographically diverse sample that included both Saudi nationals and non-Saudi expatriate denture wearers. Among participants, only 24.2% demonstrated adequate knowledge of denture hygiene. This result is consistent with the recently reported compliance rates assessed in Saudi Arabia (26, 32, 33). and internationally (9, 24, 34). This finding underscores the degree to which oral health education remains inadequately addressed in routine dental care.

Several studies linked sociodemographic factors, prosthetic history, and systemic disease to oral care compliance (24, 29, 33, 35–39). The socioeconomic gradient in knowledge identified in this study is of both clinical and policy significance. Saudi participants with high income scored more than twice as highly as non-Saudi low-income participants in oral/denture hygiene knowledge. This disparity reflects multiple disadvantages: lower educational backgrounds, language barriers, limited dental care, and reduced exposure to oral health promotion that disproportionately affect low-income non-Saudi workers. These findings align with the broader oral health equity literature (40, 41) and identify non-Saudi, low-income patients as a subgroup for targeted denture hygiene counseling in primary care. Economic constraints may limit individuals' capacity to obtain dental care services or purchase dental hygiene products. Patients in the lowest income group demonstrated the highest proportion of non-cleaning or using water rinse only to clean their dentures. However, there was no significant relation between income level and denture-cleaning method. The results suggest that denture cleaning method choice is not strongly determined by income and may instead be more closely tied to oral/denture hygiene knowledge than to socioeconomic access.

The age dimension was considered as a factor affecting oral/denture hygiene knowledge, as younger patients showed better knowledge. This study also showed that brushing the dentures was the most used cleaning method in patients in their forties; however, fewer patients aged ≥70 years used this method. This can be due to declined dexterity or general health, as the use of passive methods to clean dentures, such as soaking only or water rinsing, increases with age. Moreover, younger partial denture wearers, who have lost fewer teeth, demonstrated better knowledge and denture care behaviours. The significantly better compliance observed among young females (age < 50 years) is consistent with established gender differences in health behavior. This was shown in the literature as females generally pursue dental care earlier and more consistently compared to their male counterparts. Females consistently demonstrate higher preventive care utilization and oral health literacy across the lifespan (41). However, in this study, all genders demonstrated similar cleaning methods and frequency. Evidence indicates that females exhibit greater demands in both esthetics and function (24, 42). Females showed more effort using a brush and soaking their dentures, while males rinsed their dentures with water only.

Several studies linked systemic disease status and oral health (39, 43–45). Some demonstrated that oral hygiene care significantly influences systemic health outcomes (46). Dental disease has the potential to greatly impact systemic health, especially in relation to chronic conditions like diabetes and hypertension (39, 45). In a study that included more than 10,000 patients, those wearing dentures exhibited a higher likelihood of having diabetes and hypertension (36). In this study, we sought to choose the most commonly reported systemic disease by patients in Saudi Arabia (47, 48) to assess the relation with oral/denture care, and found no significance in the results. This suggests that, within this sample, knowledge deficits regarding denture hygiene are widespread and not specifically concentrated among medically compromised patients, implying that educational interventions addressing denture-hygiene knowledge gaps should be applied broadly across the denture-wearing population rather than targeted solely at patients with systemic comorbidity.

Studies have shown that Candida species, bacteria, and viruses are commonly isolated from the surfaces of removable dentures and denture-bearing areas (10, 49, 50). As a result, mucosal inflammation occurs in the denture-bearing area, a condition called denture stomatitis (49, 51). There is a well-established direct relationship between the quantity of plaque biofilm on dentures and the severity of denture stomatitis (7). Non-Saudi patients showed less knowledge of oral/denture hygiene; consequently, they showed a lower prevalence of stomatitis Type I and a higher prevalence of Type III, suggesting a shift toward more severe clinical types in non-Saudi patients. The relationship between non-compliance and stomatitis severity was obvious in this study, as 100% non-compliance was observed among Type III cases.

The association between cleaning frequency and denture stomatitis was obvious. Increasing cleaning frequency decreased the prevalence of denture stomatitis. Such results provide compelling clinical urgency for integrating oral examination and prosthetic hygiene assessment into primary care settings. This finding also highlights that the presence of denture stomatitis on routine examination should serve as a direct trigger for targeted hygiene counseling, documentation, and referral to further dental services.

Removing the denture during sleep was associated with less denture stomatitis occurrence (52). This agrees with our findings, as Type III stomatitis occurred only among participants who slept wearing their denture. This finding identifies nighttime removal adherence as the single most clinically actionable behavioral risk factor for stomatitis severity identified in this dataset.

Several systemic diseases are strongly associated with denture stomatitis due to altered immune responses and increased susceptibility of oral mucosa to infections (38). In this study, diabetes exhibited an intermediate pattern characterized by a notably high percentage of mild Type I cases at 33.3%, while the incidence of severe Type III cases was relatively low at 3%. The prevalence of denture stomatitis was significantly high among patients who presented with both diabetes and hypertension (36, 53). These findings are consistent with a cumulative or synergistic effect of multimorbidity of combined diabetes and hypertension found in this study on mucosal vulnerability to denture stomatitis, and indicate that systemic disease burden, rather than knowledge or behavior alone, carries independent clinical relevance for stomatitis risk stratification.

In a study evaluating the efficiency of different denture cleaning methods, brushing and soaking significantly reduced the adherence of Candida to dentures. Soaking the denture in water was found to be the least effective method (54). A numerical but not statistical difference was found in this study, as most patients with no stomatitis brushed and soaked their dentures. Interestingly, all five cleaning methods investigated in this study showed broadly comparable combined Type I–III stomatitis prevalence (55%–68%), suggesting that cleaning method alone, independent of frequency and nighttime removal habit, may be a weaker independent predictor of stomatitis than behavioral consistency (frequency, adherence) or host factors (systemic disease).

This study also showed the patient perspective on behavioral predictors of non-compliance with oral/denture care. Many removable denture wearers reported sleeping while wearing their dentures (6, 55–57). The high prevalence of nighttime denture wearing (63.3%) in this study was predominantly attributed to forgetfulness. That this behavior was driven by forgetfulness rather than intentional choice suggests that it is highly modifiable through low-intensity interventions like brief motivational counseling during primary care visits, bedside reminder cards, or simple smartphone reminders. These represent feasible, low-cost strategies that could be embedded into routine chronic disease management consultations, particularly for elderly patients attending primary care. In addition to forgetfulness, one-third of patients indicated experiencing feelings of embarrassment related to the removal of dentures during sleep. Many patients perceived a connection between the loss of teeth and the stigma associated with wearing dentures, thereby associating their dental condition with social attractiveness (58). In this study, social embarrassment regarding public denture cleaning was reported by 60% of participants. Social embarrassment is a psychosocial barrier that has received attention in the oral hygiene compliance literature (42, 59–61). Effective counseling should explicitly acknowledge this barrier and offer practical guidance (e.g., cleaning in private facilities, or implementing a pre-departure home hygiene routine).

This study reveals a gap between the institutional venues where patients received their dentures and post-operative hygiene instructions. The majority of participants (76.7%) reported receiving their hygiene instructions at a teaching hospital. However, “Dentists have no time to give instructions” was the most commonly mentioned barrier by patients to receiving comprehensive oral/denture hygiene instruction. This reflects constraints within the clinical setting and may be attributed to the high patient load present in teaching hospitals, as well as the involvement of staff and students in other educational activities. On the other hand, half of the patients who reported receiving oral/denture hygiene instruction from non-teaching settings believed that “appointments difficult to get” is the main barrier to receiving comprehensive hygiene instruction. This suggests that greater geographic or economic access constraints in this group may have shaped their care-seeking behaviour. Moreover, half of the study participants reported that “no perceived need” is also a barrier to receiving comprehensive hygiene instruction, representing a patient-centred attitudinal barrier that may be more tractable through targeted health literacy interventions. The financial barrier “expensive appointments” among this cohort of patients who predominantly have access to care publicly suggests that patients either seek private services or believe that public health institutions deter providing oral or denture care.

Studies found that many patients reported not receiving information about oral/denture care instructions or have never been informed about denture cleaning, therefore often breaching denture cleaning standards (4, 12, 62). Although only 5% of patients could not recall receiving oral/denture care instructions, this study sheds light on reported preferences for patients' educational format, including integrating denture hygiene instruction into routine primary care. Most patients reported receiving verbal instruction from dentists regarding oral and denture hygiene (24, 63). However, Mylonas et al. suggested combining verbal and written instructions with a denture hygiene kit to improve denture cleanliness (64). The strong patient preference for written instructional materials (51.7% vs. 18.3% for verbal only) has direct clinical implications. Written materials allow patients to review instructions at their own pace, share them with family or caregivers, and revisit them at moments of low compliance motivation or forgetfulness. These advantages are especially relevant for patients with lower health literacy, older patients, and non-Arabic-speaking patients receiving instructions in a second language. These findings strongly support the routine distribution of standardized, culturally adapted written denture hygiene guides at primary care settings, particularly for the elderly and non native speaking populations.

These findings collectively establish that denture hygiene non-compliance in this primary care setting is driven by a convergence of knowledge deficits, socioeconomic disadvantage, systemic comorbidity, and structural access barriers. Integration of standardised, brief denture hygiene counselling into routine primary care and chronic disease management encounters, supported by bilingual written materials and emphasising nocturnal removal as the minimum effective behaviour, represents the most pragmatic and evidence-informed strategy to reduce the unacceptably high burden of denture stomatitis in this population. Findings from this study are similar to recent international denture-hygiene research. In a single-institution cross-sectional study of 118 complete-denture wearers in Turkey, it was reported that only 40.7% demonstrated good denture hygiene despite 70.3% cleaning their dentures daily, and 56.8% removed their dentures at night (24); the comparatively high rate of reported daily cleaning alongside a still-substantial proportion of poor hygiene in that cohort mirrors the knowledge-behavior gap identified in this study. Similarly, a 2024 multicenter cross-sectional study of 217 complete and partial denture wearers in Yemen found that most participants received denture-care information from dentists (59.4%). Yet, the majority (59%) did not attend regular dental maintenance visits, and most of the patients (24.4%) used water only to clean their dentures (25). These patterns closely parallel the present findings, in which 76.7% of participants received instructions from a teaching hospital's single institutional source. Taken together with the present cohort's denture stomatitis prevalence of 65.8%, which lies within, but toward the upper end of, the previously reported global range of 15%–70% (10, 11), these cross-national comparisons suggest that inadequate denture hygiene knowledge, structurally limited reinforcement of hygiene instruction, and consequent stomatitis are shared across diverse denture-wearing populations.

Several limitations of this study should be noted. The cross-sectional design precludes causal inference between sociodemographic and behavioral predictors and both denture-hygiene knowledge and stomatitis outcomes. Moreover, participants were recruited via consecutive sampling from a single dental-intern primary care clinic affiliated with one university in Alkharj; this single-center design, combined with consecutive sampling, raises the possibility of selection bias, as patients presenting to a teaching hospital affiliated primary care clinic may differ systematically from denture wearers in the wider community. Consequently, these findings cannot be generalized to all Saudi primary care settings, and multicenter designs are needed to strengthen external validity, notwithstanding the well-recognized practical advantages of a single-center cross-sectional design for this study. Although the overall sample met the *a priori* target for estimating a single population proportion, subgroup analyses, particularly those involving non-Saudi participants (*n* = 30), were likely underpowered to detect smaller effects and did not correct for multiple comparisons; such subgroup findings should be interpreted with care. The oral/denture hygiene knowledge questionnaire was adapted from previously published instruments rather than independently re-validated in this population; no formal pilot testing of the adapted instrument was conducted, which may limit measurement precision. A key limitation of this study is that the internal consistency of the adapted oral/denture hygiene knowledge scale could not be assessed, because item-level responses to the six knowledge items were retained only as an aggregate score rather than as individual item scores; consequently, the psychometric reliability of the knowledge measure in this population remains unverified, and future studies should retain item-level data to permit formal reliability testing of the adapted instrument. The non-Arabic-speaking participants (*n* = 9) completed the questionnaire with machine-translation assistance, which may have introduced measurement variability across language groups. The questionnaire was self-administered via a standardized electronic form and was identical for every participant, which is expected to reduce interviewer-introduced variability and social-desirability pressure relative to an interview format, particularly for the compliance questions. A nurse did assist with the technical operation of the tablet for some participants, and although this assistance was intended to be limited to device navigation, we cannot fully exclude the possibility that this proximity introduced subtle social-desirability effects. In addition, only formal Arabic pronunciation was used for the device's text-to-speech, not colloquial Arabic; this may have introduced comprehension variability among participants. Another limitation of this study is that the denture stomatitis was graded by a single clinical examiner; although intra-examiner reliability was assessed and was substantial (linear-weighted kappa = 0.81), a second, independent examiner was not available to assess inter-examiner reliability, which remains a limitation of the clinical assessment methodology. Future prospective, multi-center studies incorporating larger samples, objective rather than self-reported compliance measures, formally blinded and calibrated clinical examiners, and longitudinal tracking of oral and systemic outcomes following primary care-integrated counseling interventions are warranted to address these limitations.

## Conclusion

Denture hygiene compliance is critically low among patients attending primary care settings in Alkharj, Saudi Arabia. Oral/denture hygiene knowledge adequacy was strongly predicted by nationality, age, education, and income levels. Knowledge status in turn was the dominant mediator of behavioural compliance: patients with adequate knowledge were more likely to clean twice or more daily.

Denture stomatitis prevalence was high, particularly in patients with reduced cleaning frequency; however, its severity increased with age and was substantially amplified by systemic comorbidity. Patients who consistently removed their dentures at night were twice as likely to remain stomatitis-free compared with habitual retainers, identifying nighttime removal as the most actionable behavioural target for clinical counselling.

Multiple structural and attitudinal barriers impeded access to hygiene instruction: clinician time constraints, appointment costs, difficulty obtaining appointments, and low perceived need for professional guidance were each endorsed by more than one-third of participants. Patients expressed a clear preference for written or combined written-and-verbal instructional formats for oral/denture hygiene instructions.

## Data Availability

The original contributions presented in the study are included in the article/Supplementary Material, further inquiries can be directed to the corresponding author/s.
