# Supplementary material for: Denture hygiene knowledge, cleaning practices, and denture stomatitis among primary care patients in Alkharj, Saudi Arabia: compliance predictors, barriers and educational preferences: a cross-sectional study
Source: Front Med (Lausanne). 2026 Sep 23;13:1927858. doi: 10.3389/fmed.2026.1927858 (PMC13642781; doi:10.3389/fmed.2026.1927858)
Supplement: Supplementary file 1 [file Table1.docx]

**Research Consent Form and Data Collection Form**

**PURPOSE OF STUDY (RESEARCH)**

You are kindly invited to participate in a research study, the title of which is:

Denture Hygiene Knowledge, Cleaning Practices, and Denture Stomatitis Among Primary Care Patients in Alkharj, Saudi Arabia.

The aim of this study is as follows to determine oral and denture hygiene knowledge, frequency of cleaning, cleaning method, and denture stomatitis among primary care attendees in Alkharj, Saudi Arabia, based on 1) sociodemographic factors; 2) prosthetic history; and 3) systemic disease. Identify behavioral predictors of non-compliance and evaluate patient-reported barriers and educational format preferences to inform the integration of denture hygiene counseling into routine primary care encounters.

**PROCEDURE FOR THE RESEARCH STUDY**

By participating in this study, you will need to answer a few questions about your sociodemographic data, prosthetic history, and systemic disease. Then, the dentist will perform a dental examination using a mouth mirror and will examine your denture too.

**RISKS OF PARTICIPATING IN THE RESEARCH**

There are no risks in participating in this study mentioned above.

**COST OF PARTICIPATION**

No cost for participation in this study.

**BENEFIT OF THE RESEARCH**

This study will help us understand denture hygiene knowledge, cleaning practices, and denture stomatitis prevalence. Identify behavioral predictors of non-compliance and evaluate patient-reported barriers and educational format preferences to inform the integration of denture hygiene counseling among primary care patients in Alkharj, Saudi Arabia.

**PAYMENT FOR PARTICIPATING**

No payment for participating.

**CONFIDENTIALITY**

All your information will be anonymized before data analysis, and none of your personal data will be reported or stored.

I have read the content of this document, and/or it has been fully explained to me. All of my questions were answered satisfactorily, and I have been given a copy of the document. I voluntarily agree/consent to take part in this study.

**SUBJECT’S FULL NAME	Date**

**SUBJECT’S SIGNATURE**

*Thumb print (if applicable)*

**Part 1: *Select what applies to you:***

**Your Nationality?**

- Saudi
- Non-Saudi

**Age:** ____ years

**Gender?**

- Female
- Male

**Do you speak Arabic fluently?**

- Yes
- No

**Your Education level**

- None/primary
- Secondry
- University/postgraduate

**Your Income level**

- Low (< SAR 4,000)
- Moderate (SAR 5,000–10,000)
- High (> SAR 10,000)

**Do you have any systemic disease:**

- None
- Hypertension
- Diabetes mellitus
- Combined diabetes and hypertension
- other systemic conditions

**When was your last dental visit?**

- Less than 6 months
- 6–12 months
- 1–2 years
- More than 2 years

**How many time you clean your denture per day:**

- 0
- 1
- 2
- 3 or more

**How do you clean your denture:**

- No cleaning
- Water rinse only
- Soak only
- Brushing only
- Combined brushing and soaking
- Combined brushing/soaking or Ultrasonic

**Do you remove your denture when you sleep at nighttime**

- Yes
- No

**If you select no, why do you keep your denture in your mouth when you sleep?**

- No need to remove it
- You sleep better with your denture
- You forget to remove it
- You are embarrassed to sleep without it

**Do you remove and clean your denture in public if you need to?**

- Yes
- No

**If you select no, why do you not remove and clean your denture in public?**

- You do not wear your denture in public
- You do not need to remove your denture to clean it
- You are embarrassed to remove your denture in public

**Do you feel you can clean your dentures properly?**

- Yes
- No

**Has a dentist or his/her staff ever shown you or told you how to clean dentures?**

- Yes
- No

**What is your preferred method of receiving instruction about oral/denture hygiene by your dentist?**

- Written instructions
- Verbal instructions
- Both written and verbal instructions
- Video / Digital media

**Do you think there is (are) barriers to receiving oral/denture hygiene instructions (Multiple Responses Permitted) from your dentist?**

- There is no need for professional instructions
- Fear of dental appointments
- Expensive dental appointments
- Dental appointments are difficult to get
- Dentists have no time to give instructions
- I do not have time to book an appointment to receive professional instructions
- Other reasons

**Part 2: For the dentist to fill**

**Denture type**

- Complete denture
- Conventional partial denture

**Missing teeth pattern (select all applicable)**

- Anterior teeth
- Premolar teeth
- Molar teeth

**Evaluate denture bearing area using Newton's clinical grading system**

- **Type I:** Localized simple inflammation or pinpoint hyperemia.
- **Type II:** Generalized simple inflammation erythema.
- **Type III:** Granular inflammation (inflammatory papillary hyperplasia).
